# Characteristics of retinal vascularization in reactivated retinopathy of prematurity requiring treatment and clinical outcome after reinjection of ranibizumab

**DOI:** 10.1038/s41598-024-66483-2

**Published:** 2024-07-08

**Authors:** Ji Hye Jang

**Affiliations:** Department of Ophthalmology, School of Medicine, Keimyung University, Daegu, 42601 Republic of Korea

**Keywords:** Ranibizumab, Reactivation, Retinopathy of prematurity, Retreatment, Health care, Medical research

## Abstract

This study aimed to determine whether the state of retinal vascularization after anti-vascular endothelial growth factor (anti-VEGF) injection can help predict the risk of reactivated retinopathy of prematurity (ROP) requiring treatment and whether repeated ranibizumab injection will be effective in such cases. We retrospectively reviewed 24 infants (43 eyes) who received ranibizumab monotherapy between January 2021 and December 2022. All eyes were classified as having non-retreated ROP or retreated ROP. The state of ROP at the time of treatment, the time required for resolution of plus disease, and the extent of vascularization at 4 and 8 weeks after treatment were analyzed. Extent of temporal retinal vascularization was measured with serial fundus images using disc-fovea distance (DF) unit and disc diameter (DD). Reactivated ROP requiring treatment occurred in six infants (25.0%) and ten eyes (23.3%) after ranibizumab treatment. The mean retreatment interval was 9.0 ± 3.3 weeks (range 4–16). In the retreated ROP group, the time required for the resolution of plus disease after primary injection was longer compared to the control group (13.3 days vs 5.2 days), with a mean ROP regression time of 3.4 weeks. All eyes in the retreated ROP showed retinal vascularization < 0.5 DF from the original site at 4 weeks after injection. In 90% of cases with retreated ROP, the extent of vascularization at 8 weeks after injection was within 1 DF from the original ROP site, and all cases showed reactivation in the posterior Zone II area. The extent of retinal neovascularization in the retreated group was an average of 0.7 DD (vs 1.7 DD) and 1.3 DD (vs 3.3 DD) at 4 and 8 weeks after injection, respectively. After ranibizumab retreatment, only one reactivated case with vitreous traction progressed to focal retinal detachment, while all other cases regressed with peripheral vascular development. The continuation of delayed retinal blood vessel development after ≥ 8 weeks may indicate a high likelihood of reactivated ROP requiring treatment. In the absence of vitreous traction, ranibizumab reinjection is likely to be effective in treating reactivated ROP requiring treatment.

## Introduction

Retinopathy of prematurity (ROP) is a retinal proliferative vascular disease that occurs only in premature infants with incompletely vascularized retina. It begins with the arrest or delay in physiological retinal vascular development and progresses to abnormal angiogenesis due to ischemia and high levels of various angiogenic factors, leading to tractional retinal detachment^1,2^.

Vascular endothelial growth factor (VEGF)^3,4^, one of the angiogenic factors, plays an important role in retinal vascular development. High levels of VEGF in the vitreous cavity are closely related to the development of ROP. The BEAT-ROP study^5^ and the RAINBOW study^6^ demonstrated the effectiveness of intravitreal injection of anti-VEGF agents in treating ROP. Recent years have witnessed a paradigm shift in the treatment of ROP from laser treatment to anti-VEGF injection^7^. Anti-VEGF injection offers several advantages over laser treatment such as eliminating the need for general anesthesia, providing a rapid clinical response, and permitting vascularization into the immature retina without causing permanent retinal damage^8^. Therefore, for zone I ROP or aggressive ROP, anti-VEGF treatment is more effective than laser treatment^9,10^.

After anti-VEGF injection, plus disease usually begins to regress and retinal vascularization begins to progress^5,6,11^. After complete regression of the plus disease, reactivation is likely to occur if retinal blood vessels do not form properly^11^. Therefore, determining the clinical characteristics of regression and reactivation after anti-VEGF treatment is imperative.

Reactivation after treatment with ranibizumab is known to occur more frequently and much earlier than with bevacizumab^12–16^. In a recent US multicenter study^15^, eyes receiving ranibizumab had a higher retreatment rate compared to eyes receiving bevacizumab (58% vs 37%, *p* < 0.001). The high frequency of recurrence after ranibizumab administration may be related to its shorter half-life compared to bevacizumab^13^. Therefore, close follow-up of eyes injected with ranibizumab is important until the formation of retinal blood vessels in the periphery or stabilization of active disease.

However, the key aspects that need to be assessed during follow-up and the optimal treatment approach in case of recurrence are not well standardized. Therefore, we aimed to identify the conditions that are most likely to lead to reactivated ROP requiring treatment after ranibizumab injection, the optimal approach to post-treatment follow-up, and whether repeat ranibizumab treatment is effective in such cases.

## Materials and methods

**Figure 1:**
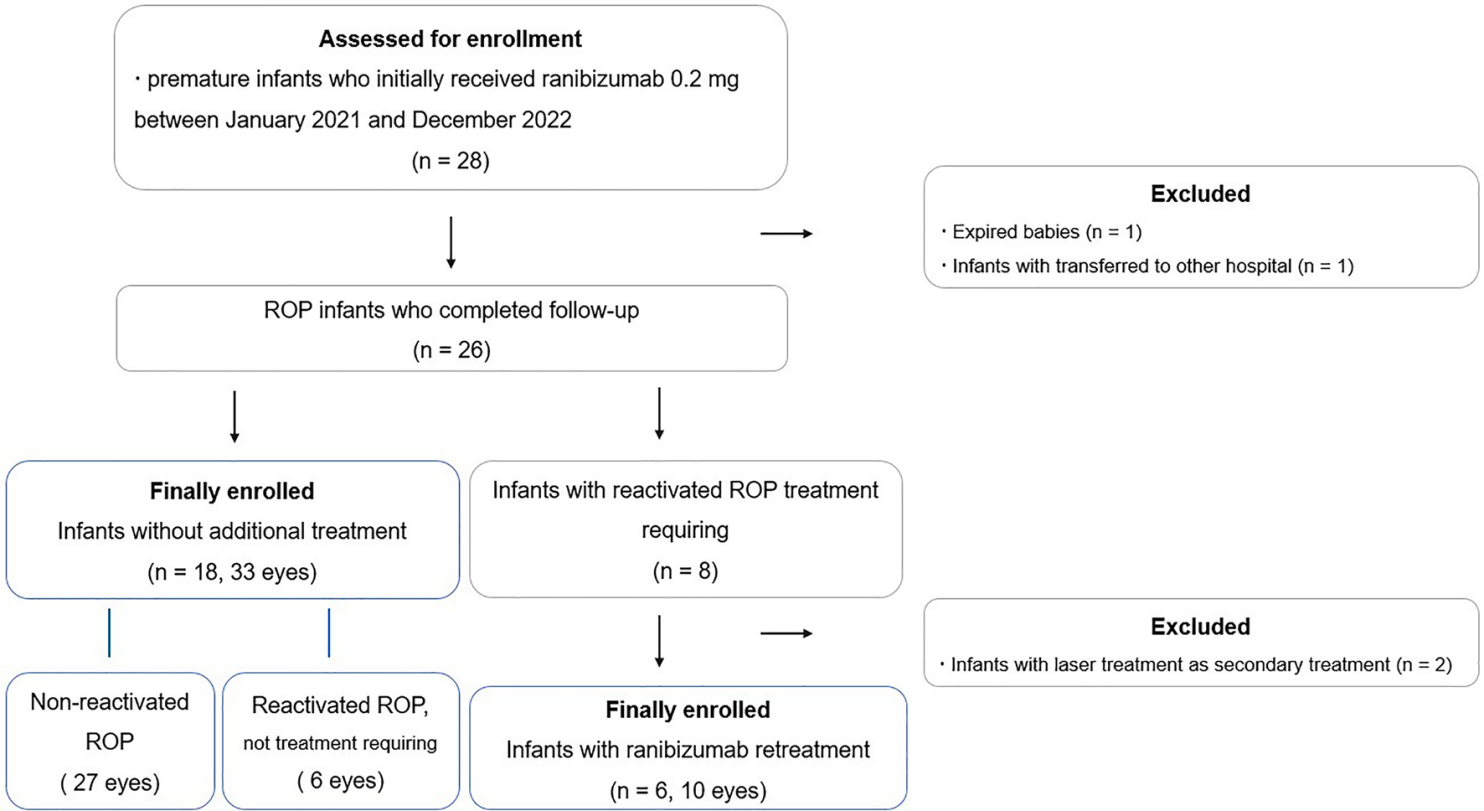
Flow diagram illustrating the enrollment process.

This was a single-center retrospective case series study. Ethical approval was obtained from the Keimyung University Hospital Institutional Review Board (No. 2023-12-001) in accordance with the principles of the Declaration of Helsinki. We retrospectively analyzed the medical records of premature infants who initially received ranibizumab 0.2 mg between January 2021 and December 2022 and completed follow-up until 70 weeks postmenstrual age (PMA, 70 ± 3 weeks). The exclusion criteria were as follows: 1) infants who received combination therapy (laser treatment and anti-VEGF injection) or vitreoretinal surgery as the primary treatment for ROP; 2) after reactivation of ROP, treatment other than ranibizumab reinjection was administered as secondary treatment; 3) infants who died before completion of follow-up, infants with incomplete follow-up or follow-up loss after injection, and infants transferred to other hospitals. However, if the infant moved to another hospital after ranibizumab reinjection and underwent surgery as a third treatment, it was considered a treatment failure (Fig. 1).

### ROP follow-up protocol after ranibizumab injection

All eye examinations and treatments were performed by a single experienced retina specialist (JJH). Fundus examination was performed using an indirect ophthalmoscope, and fundus photographs were obtained using a wide-angle fundus camera (RETCAM, Clarity Inc., Pleasanton, CA, USA). The features of ROP were recorded at each examination based on the guidelines published by ICROP3 (International Committee for the Classification of Retinopathy of Prematurity, 3rd edition)^17^.

Infants with aggressive ROP or type 1 ROP were treated with intravitreal ranibizumab within 48 h, if possible. Informed consent was obtained from the patient’s parents before injection treatment. In the neonatal intensive care unit, ranibizumab was injected intravitreally at 0.2 mg/0.02 mL (same dose as administered in the RAINBOW study) under topical anesthesia.

Infants were examined on days 3 and 7 after injection and weekly follow-up was continued for 8 weeks or until 45 weeks PMA. If there was no ROP reactivation up to 8 weeks after injection, follow-up exams were performed at 2 week intervals for at least 55 weeks PMA, and thereafter at 3-week intervals until 70 weeks PMA.

Reactivation of ROP was determined according to ICROP3^17^, and vascular abnormalities such as the reappearance of plus disease spectrum or the formation of a new demarcation line were considered the first symptoms of reactivation. Reactivated ROP requiring treatment was defined as a reactivated stage 2 + or greater lesion at the original site or at the advanced edge. Retreatment was performed with ranibizumab at the same dose as the first injection Follow-up exams were performed at 1 week intervals until 4 weeks after reinjection and at 2 or 3 week intervals thereafter until PMA 70 weeks.

### ROP without ranibizumab retreatment vs ROP with ranibizumab retreatment

All eyes were divided into two groups depending on whether additional ranibizumab treatment was needed. The ROP without retreatment group included ROP that regressed well after the first ranibizumab injection and reactivated ROP that did not require additional treatment. The retreated ROP group included reactivated ROP requiring treatment with ranibizumab as second-line treatment.

Demographic data such as sex, gestation age (GA), and birth weight (BW) were collected and analyzed. Ophthalmological data were collected and analyzed per eye rather than per subject; PMA at the primary treatment, ROP type (type 1 vs aggressive), ROP zone and stage at treatment, time of plus disease disappearance, duration of disease regression after treatment, presence of reactivation, the extent of temporal retinal vascularization at 4 weeks and 8 weeks after primary injection, time taken for peripheral vascular progression to form up to Zone II.

**Figure 2:**
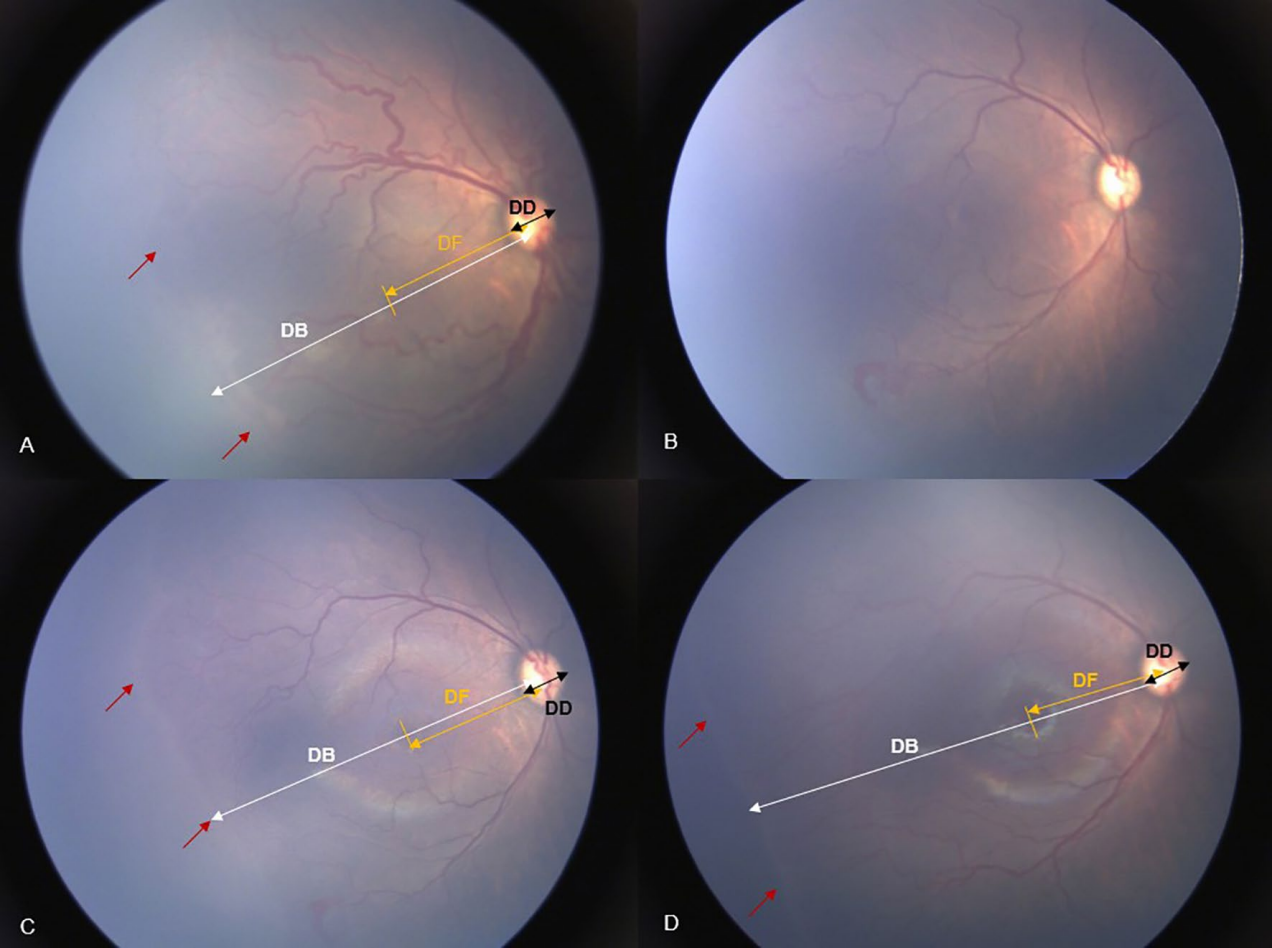
The two measurements of method of extent of temporal retinal vascularization using serial fundus images in reactivated retinopathy of prematurity (#6 ranibizumab retreated case). (**A**) Fundus image of the right eye at the time of primary ranibizumab injection; (**B**) Fundus image at 1 week after ranibizumab injection; (**C**) Fundus image at 4 weeks after ranibizumab monotherapy showing advanced edge of retinal vascularization (red arrow); (**D**) Fundus image at 8 weeks following ranibizumab monotherapy showing advanced edge of retinal vascularization (red arrow). One method is a calculating the ratio between the distance from the center of the optic disc to the temporal edge passing through the fovea (DB, white double arrow) and the distance from the center of the optic disc to the center of the fovea (DF, orange double arrow) at the time of primary injection, 4 weeks after injection, and 8 weeks after injection. Another methods is using disc diameter (DD, black double arrow) unit, calculating the ratio of DB and DD before treatment, at 4 weeks after injection, and at 8 weeks after injection.

The extent of temporal retinal vascularization was measured in two ways using serial retinal RETCAM™ images of medium-to-high quality to help identify the location of temporal retinal vascular edges. The first method, described by Lorenz et al.^18^, was to use disc-fovea (DF) units defined as the distance from the center of the optic disc to the center of the fovea. The foveal center was determined based on the foveal reflection of the photograph. The length of the temporal vascularized retina was defined as the distance from the center of the optic disc to the temporal edge passing through the fovea, denoted as the disc-border (DB). The ratio between DB and DF was calculated before treatment, at 4, 8 weeks after injection, and the difference was defined as the extent of retinal vascularization. Another method, proposed by Bayramoglu et al.^19^, was to use disc diameter (DD) unit. The ratio of DB and DD was calculated before treatment, at 4, 8 weeks after injection and the difference was defined as the extent of retinal vascularization (Fig. 2).

Ranibizumab-retreated eyes were further examined for data on the timing of retreatment, characteristics of reactivated ROP status (zone and stage), site of recurrence, and outcomes after ranibizumab reinjection.

### Statistical analysis

Continuous variables were expressed as mean ± standard deviation, and the mean difference between the two groups was compared using an independent t- test. Categorical variables were expressed as frequency and percentage, and the difference in proportions between the two groups was compared using the chi-square test. The statistical analysis was performed using SAS 9.4 (Copyright (c) 2002–2012 by SAS Institute Inc., Cary, NC, USA.); *p-*values < 0.05 were considered indicative of statistical significance.

## Results

Twenty-four infants (43 eyes) were included in this study. Reactivated ROP requiring treatment occurred in 6 of 24 (25%) infants and 10 of 43 eyes (23.3%) that received the first ranibizumab injection. For Type 1 ROP, additional ranibizuamb injections were performed in 6 eyes, including 3 eyes with Zone I ROP and 3 eyes with posterior Zone II 3 + ROP. Additionally, 4 out of 10 aggressive ROP cases received ranibizumab reinjection. In posterior zone II 3 + ROP, 6 of 26 eyes developed reactivated ROP but did not require treatment (Table 1).

**Table 1:** Demographics of ROP infants receiving ranibizumab 0.2 mg as primary treatment.

Parameters	Retreated ROP	Non-retreated ROP	*p*-value
Number of Infants (n, %)	6 (25.0%)	18 (75.0%)	–
Eyes of infants (n, %)	10 (23.3%)	33 (76.7%)	–
Male: Female (n : n)	3 : 3	11 : 7	0.432
Gestational age (weeks, mean ± SD, [min, max])	26.6 ± 0.2 (23, 29)	26.2 ± 2.1 (23, 30)	0.911
Birth weight (grams, mean ± SD, [ min, max])	712.0 ± 132.5 (610, 950)	818.2 ± 205.7 (540, 1540)	0.133
PMA at the primary treatment (weeks, mean ± SD, [min, max])	35.1 ± 1.2 (33, 37)	38.5 ± 6.0 (35, 47)	< .001*
Treated eye at the primary treatment			-
One eye only (n, %)	2 (33.3%)	3 (16.7%)	
Both eyes (n, %)	4 (66.7%)	15 (83.3%)	
ROP type at the primary treatment			0.851
Type 1 ROP (eyes, %)	6 eyes (60.0%)	23 eyes (69.7%)	
Zone I ROP	3 eyes (30.0%)	0 eyes	
Posterior Zone II 3 + ROP	3 eyes (30.0%)	23 eyes (69.7%)	
Aggressive ROP (eyes, %)	4 eyes (40.0%)	10 eyes (30.3%)	

*ROP* retinopathy of prematurity, *PMA* postmenstrual age.

*Statistically significant by independent t-test or the chi-square test.

In the retreated ROP group, there was an equal proportion of males and females and the mean GA and BW were 26.6 ± 0.2 weeks and 712.0 ± 132.5 g, respectively. In the non-retreated ROP group, 61.1% were male and the mean GA and BW were 26.2 ± 2.1 weeks and 818.2 ± 205.7 g, respectively. The mean PMA at first ranibizumab monotherapy in the retreated ROP group was significantly lower than that in the non-retreated ROP (35.1 ± 1.2 weeks vs 38.5 ± 6.0 weeks; *p* < 0.001) (Table 1).

All cases in the retreated ROP group showed resolution of plus disease more than 1 week after the first injection of ranibizumab (average: 13.3 ± 12.7 days). In the non-retreated ROP group, the mean time from the first injection of ranibizumab to resolution of plus disease was 5.2 ± 1.7 days, with 57.6% of cases showing resolution of plus disease within 1 week and 42.4% showing resolution after 1 week. The time taken for ROP to regress was 3.4 ± 2.7 weeks in the retreated ROP group and 1.9 ± 1.2 weeks in the non-retreated ROP group.

All eyes in the retreated ROP group showed temporal retinal vascularization of < 0.5 DF from the original site at 4 weeks after the first injection, with a mean of 0.2 ± 0.2 DF, a mean of 0.7 ± 0.6 DD. In the non-retreated ROP group, the mean extent of vascularization from the original ROP location at 4 weeks after the first injection was 0.5 ± 0.3 DF and 1.7 ± 0.9 DD; it was < 0.5 DF in 33.3%, 0.5 to 1 DF in 54.6%, and > 1 DF in 12.1%, respectively.

The mean extent of vascularization at 8 weeks after primary injection was 0.5 ± 0.3 DF, 1.3 ± 1.2 DD in the retreated ROP group and 1.1 ± 0.3 DF, 3.3 ± 1.2 DD in the non-retreated ROP group (*p* < 0.001). In the retreated ROP group, the distribution of eyes with retinal vascularization within 0.5 DF, eyes with retinal vascularization between 0.5 and 1 DF, and eyes with retinal vascularization more than 1 DF at 8 weeks after injection was 4 eyes (40%), 5 eyes (50%), and 1 eye (10%), respectively. The corresponding distribution in the non-retreated ROP group was 4 eyes (40%), 5 eyes (50%), and 1 eye (10%), respectively. The mean time of the formation of retinal vessels up to zone II in the reactivated ROP group was significantly longer than that in the non-reactivated ROP group (18.9 ± 5.2 weeks vs 9.4 ± 2.8 weeks; *p* < 0.001) (Table 2).

**Table 2:** Primary treatment response between non-retreated and retreated ROP groups.

Parameters	Retreated ROP (n = 6, 10 eyes)	Non-treated ROP (n = 18, 33 eyes)	*p*-value
Plus disease disappearance (days, mean ± SD)	13.3 ± 12.7	5.2 ± 1.7	0.089
< 1 week	0	19 eyes	0.002*
≥ 1 week	10 eyes	14 eyes	
ROP regression after primary injection (weeks, mean ± SD)	3.4 ± 2.7	1.9 ± 1.2	0.129
Extent of temporal vascularization at 4 weeks after primary injection (DF, mean ± SD)	0.2 ± 0.2	0.5 ± 0.3	< .001*
< 0.5 DF from original location of ROP (n, %)	10 eyes (100%)	11 eyes (33.3%)	0.001*
0.5 to 1 DF from original location of ROP (n, %)	–	18 eyes (54.6%)	
≥ 1 DF from original location of ROP (n, %)	–	4 eyes (12.1%)	
Extent of temporal vascularization at 4 weeks after primary injection (DD, mean ± SD)	0.7 ± 0.6	1.7 ± 0.9	0.002*
Extent of temporal vascularization at 8 weeks after primary injection (DF, mean ± SD)	0.5 ± 0.3	1.1 ± 0.3	< .001*
< 0.5 DF from original location of ROP (n, %)	4 eyes (40%)	4 eyes (12.1%)	0.001*
0.5 to 1 DF from original location of ROP (n, %)	5 eyes (50%)	4 eyes (12.1%)	
≥ 1 DF from original location of ROP (n, %)	1 eye (10%)	25 eyes (75.8%)	
Extent of temporal vascularization at 8 weeks after primary injection (DD, mean ± SD)	1.3 ± 1.2	3.3 ± 1.2	< .001*
Vascular progression up to Zone II (weeks, mean ± SD)	18.9 ± 5.2	9.4 ± 2.8	< .001*

*SD* standard deviation, *ROP* retinopathy of prematurity, *DF* distance units from the disc to fovea, *DD* disc diameter.

*Statistically significant by independent t-test or the chi-square test.

Table 3 shows details of infants with ranibizumab retreated ROP. The timing of retreatment was 39–53 weeks PMA, with an average time of 9.0 ± 3.3 weeks after the first injection. All cases showed reactivation in the posterior Zone II area, including the advanced edge or the original lesion. The success rate of the second ranibizumab injection was 90%. All patients except infant 1 regressed to peripheral vascular development. Infant #1 had a reactivated lesion with vitreous traction, which progressed to tractional retinal detachment after the second injection, and surgery was performed.

**Table 3:** Details of reactivated ROP infants following ranibizumab reinjection.

Infants	GA (weeks + days)	BW (grams)	At 1st ranibizumab injection				At 2nd ranibizumab injection					
			PMA (weeks + days)	Eyes	ROP type	Zone	PMA (weeks + days)	Eyes	retreatment interval (weeks)	Zone	Recurrence sites	Outcome
1	27 + 5	950	34 + 2	OU	Type 1	Posterior Zone II	39 + 2	OD	5	Posterior Zone II	Advanced edge	4B
2	23 + 5	560	33 + 3	OU	Type 1	Posterior Zone II	42 + 3	OU	9	Posterior Zone II	Original	Regressed
3	25 + 2	840	34 + 3	OU	Aggressive	Zone I	44 + 3	OU	10	Posterior Zone II	Advanced edge	Regressed
4	24 + 4	640	35 + 0	OU	Aggressive	Zone I	43 + 0/45 + 0	OS/OD	8/11	Posterior Zone II	Advanced edge	Regressed
5	29 + 1	610	37 + 6	OU	Type 1	Posterior Zone II	53 + 6	OS	16	Posterior Zone II	Original	Regressed
6	25 + 6	740	36 + 0	OU	Type 1	Posterior Zone II	40 + 0/44 + 0	OS/OD	4/8	Posterior Zone II	Advanced edge	Regressed
Range or mean values	23—29	610—950	33—37				39—53	10 eyes	9.0 ± 3.3			

## Discussion

This study aimed to identify the retinal vascular conditions associated with the reactivated ROP requiring treatment after ranibizumab monotherapy, the key aspects that should be monitored during follow-up, and whether repeat ranibizumab treatment is effective in reactivated ROP.

The study findings are summarized as follows: (1) In all infants with reactivated ROP requiring treatment, it took more than 1 week for the resolution of plus disease. (2) After the first ranibizumab injection, retina vascular formation in the peripheral area was slower in the ranibizumab retreated ROP group than in the non-retreated ROP group. Eight weeks after injection, 90% of the ranibizumab retreated ROP group showed retinal vascularization within 1 DF from the original lesion. (3) In reactivated ROP requiring treatment, all recurrent lesions occurred in posterior zone II, and the formation of retinal blood vessels in zone II occurred at an average of 18.9 ± 5.2 weeks after the first injection. (4) In the absence of vitreous traction, reinjection of ranibizumab is effective in the treatment of reactivated ROP requiring treatment.

The reported recurrence rate after ranibizumab treatment ranges from 4.3% to 52%^20^. In the RAINBOW study^11^, 22 eyes (15%) in the ranibizumab 0.2 mg group and 26 eyes (17%) in the ranibizumab 0.1 mg group received additional treatment due to ROP recurrence. The RAINBOW study^6^ recommends the use of 0.2 mg ranibizumab, but in real-world settings, ranibizumab is used in various doses from 0.15 mg to 0.3 mg^11,15,21^. A US multicenter study^15^ found no significant difference in the retreatment rate between the low-dose and high-dose ranibizumab groups. In this study, the retreatment rate due to recurrence of ROP treated with 0.2 mg injection of ranibizumab was approximately 25%.

The risk period for recurrence after intravitreal bevacizumab monotherapy is from 45 to 55 weeks PMA, which is the critical 10-week “recurrence window”; in rare cases, recurrence may occur up to 65 weeks PMA^21^. In the BEAT-ROP study, the mean time to recurrence after bevacizumab monotherapy was 16.0 ± 4.6 weeks^5^.

There are differences in the recurrence rate and duration of ROP depending on the anti-VEGF agent used, which may be related to their different pharmacokinetic properties. In the study by Chan et al.^22^, ROP recurred at an average of 7.6 weeks after the administration of ranibizumab. Gunay et al.^16^ reported that recurrence occurred at an average interval of 8.75 ± 1.5 weeks after treatment with ranibizumab, whereas the average interval was considerably longer after treatment with bevacizumab (14 ± 2.65 weeks). According to a meta-analysis of ROP study^21^, retreatment in the intravitreal ranibizumab group (9.29 ± 4.47 weeks) was earlier than that in the bevacizumab group (11.36 ± 4.31 weeks) or aflibercept group (12.96 ± 2.24 weeks). Consistent with the previous studies, the time of retreatment in the present study was 39 to 53 weeks PMA, and the average retreatment interval after the first injection was 9.0 ± 3.3 weeks (range 4–16).

The typical clinical course after anti-VEGF injection is that the plus disease typically begins to regress within 1 week and the ROP lesion regresses in 2 to 4 weeks^11,17^. In the present study, it took more than 1 week for the plus disease to disappear in all cases in the reactivated ROP requiring treatment. Moreover, the time required for ROP regression was longer in the reactivated ROP group than in the non-reactivated ROP group (3.4 vs 2.0 weeks). This may be due to higher pre-injection VEGF concentrations in cases of reactivated ROP requiring treatment. In our study, reactivation requiring more treatment occurred in zone I ROP and aggressive ROP than in posterior zone II 3 + , so the status of ROP before treatment may also be an important factor in retreatment.

ROP treated with anti-VEGF injections has a higher risk of recurrence when retinal vascularization beyond the avascular area is very slow or halted and the ischemic retinal area becomes very large over time^23–25^. A question arises: To what extent should retinal blood vessels form after anti-VEGF injection to reduce the risk of ROP recurrence? This is an important issue in follow-up after anti-VEGF therapy. The area from the optic disc to 2 DF corresponds to Zone I, the area from 2 to 3 DF corresponds to posterior Zone II, and the area from 3 to 4 DF corresponds to Zone II^18^. Because the DF/DD ratio decreased most rapidly during infancy, Bayramoglu^19^ recommended using DD units instead of DF units when measuring the length of retinal vascularization. Therefore, in this study, the extent of temporal retinal vascularization was measured in two ways using both DF and DD units.

Mintz-Hittner et al.^26^ reported that the anterior extent of retinal vascularization was mean 4.48 D in non-recurrent ROP and 1.76 DD in recurrent ROP after bevacizumab monotherapy and the rate of vascularization was 0.23 DD/week in non-recurrent eyes and 0.11 DD/weeks in recurrent eyes. In this study, the extent of temporal retinal vascularization in the ranibizumab retreatment group was approximately 40% of that in the non-retreatment group (mean 0.7 DD vs 1.7 DD at 4 weeks after primary injection, mean 1.3 DD vs. 3.3 DD at 8 weeks after primary injection in those with versus without retreatment, respectively).

Wu et al.^27^reported that retinal vascularization of 4 DF from the optic disc to the temporal retina and 3.3 DF to the nasal retina after ranibizumab monotherapy can be considered a safe indicator of reactivation after ranibizumab therapy. In this study, the extent of vascularization at 4 weeks after primary ranibizumab injection in the retreated ROP group was less than 0.5 DF from the original lesion in all cases, and the extent of vascularization at 8 weeks after injection was less than 1 DF in 90% of cases. Additionally, all recurrence areas were in the posterior Zone II, including the advanced edge or the original lesion. Therefore, if retinal vascularization is safely formed up to Zone II, the risk of reactivated ROP requiring treatment can be considered low.

Bayramoglu^19^ reported that in bevacizumab-treated eyes, the FD/DD ratio decreased from 4.20 at 36 weeks of PMA to 3.56 at 69 week of PMA. Similarly, in our study, in eyes treated with ranibizumab, the FD/DD ratio decreased from 4.30 to 3.70 during 8 weeks after primary treatment (Supplement 1). Whether evaluated by DF unit or DD unit, it is consistent that reactivation ROP requiring treatment occurs frequently when retinal vascularization is delayed. In this study, it was considered more useful measure the extent of retinal vascularization using DF because the DF unit was the standard for distinguishing zones in ICROP3.

It is important to assess whether ROP has regressed well or is at risk of recurrence until 55 weeks of PMA, but continuous checkups thereafter are a burden for both the baby and their guardian. So, what is the optimal duration of follow-up retinal examination for premature infants who have received anti-VEGF injection treatment? Toy et al.^23^ suggested that ROP should be monitored until retinal vessels have formed within 2 DD of the ora serrata.

The present study suggests that it is important to carefully check the following aspects during follow-up after ranibizumab administration. First, did the plus disease disappear within 1 week after the first injection? Second, did the ROP lesion regress within 4 weeks? In most cases, when regression occurs within 2 weeks, recurrence is unlikely to occur. Additionally, if regression does not occur within 4 weeks, it can be considered as incomplete regression. Third, have more than 1 DF of vascular progression formed at 8 weeks after injection? Fourth, were retinal blood vessels formed up to zone 2 after injection?

Most cases of ROP require only one or two anti-VEGF injections^11,15,26,28^ A third injection is rarely required. Martínez-Castellanos et al.^29^ recommended that anti-VEGF injection should be repeated if ROP worsens due to inadequate technique, if elevation of ridge with new vessels is observed, and if flat new blood vessels are observed; however, vitrectomy should be performed if vitreous traction is observed above the ridge. In the Korea multicenter study^10^, laser treatment was preferred over anti-VEGF reinjection in cases requiring additional treatment. However, when anti-VEGF injections were selected, they tended to be administered at a dose equal to or higher than the first injection. In the present study, Infant #1 had a reactivated lesion with vitreous traction (blue arrow) that progressed to focal retinal detachment after reinjection. Therefore, in cases with vitreous traction over a ridge or extraretinal vascular proliferation, re-injection of ranibizumab may lead to tractional retinal detachment.

Some limitations of this study should be considered. This was a retrospective singer-center study. Only extent of temporal retinal vascularization was analyzed. In addition, we did not use the vascular severity score (an objective indicator of the changes in plus disease) and fluorescein angiography (to delineate the retinal vascular abnormalities and peripheral retinal state). However, unlike other studies, this study provides valuable insights for the follow-up of ROP patients after ranibizumab monotherapy. It is important to check whether regression is occurring up to 4 weeks after injection and to check whether retinal vascularization is well formed at least 1DF from the original lesion at 4 to 8 weeks. Moreover, this study is meaningful in that few studies have reported clinical outcomes after re-injection of ranibizumab.

In conclusion, we performed serial fundus photography and fundus examination to identify the characteristics of retinal blood vessels associated with reactivated ROP requiring treatment. Our findings suggest that reactivated ROP requiring treatment is more likely to occur if retinal vascular development is slow even more than 8 weeks after injection. In the absence of vitreous traction, ranibizumab reinjection is considered effective in treating recurred ROP. However, further fluorescein angiography studies will help standardize the follow-up guidelines after anti-VEGF therapy and treatment protocols for reactivated ROP.

### Supplementary Information

Supplementary Information.

## Data Availability

The datasets used or analyzed during the current study are available from the corresponding author on reasonable request.
