# Supplementary material for: Characteristics of retinal vascularization in reactivated retinopathy of prematurity requiring treatment and clinical outcome after reinjection of ranibizumab
Source: Sci Rep. 2024 Jul 8;14:15647. doi: 10.1038/s41598-024-66483-2 (PMC11231209; doi:10.1038/s41598-024-66483-2)
Supplement: Supplementary file 1 — Supplementary Information. [file 41598_2024_66483_MOESM1_ESM.docx]

**Supplement 1.** The ratio of disc- fovea distance unit and disc diameter unit by age

|  | DF/DD ratio at primary injection treatment  ( mean 35 ~ 38 weeks PMA) | DF/DD ratio at 4 weeks after primary injection | DF/DD ratio at 8 weeks after primary injection |
| --- | --- | --- | --- |
| Eyes with retreated ROP | 4.42 ± 0.78 | 3.73 ± 0.43 | 3.65 ± 0.33 |
| Eyes with non-treated ROP | 4.10 ± 0.43 | 3.80 ± 0.42 | 3.80 ± 0.38 |
| *p*-value^†^ | 0.3261 | 0.7380 | 0.4510 |

DF: the distance form optic disc to foveal center; DD: disc diameter

† independent t-test
